# Antagonistic Activity of Bacteria Isolated from the *Periplaneta americana* L. Gut against Some Multidrug-Resistant Human Pathogens

**DOI:** 10.3390/antibiotics10030294

**Published:** 2021-03-11

**Authors:** Asmaa Amer, Basma Hamdy, Dalia Mahmoud, Mervat Elanany, Magda Rady, Tahani Alahmadi, Sulaiman Alharbi, Sara AlAshaal

**Affiliations:** 1 Department of Entomology, Faculty of Science, Ain Shams University, P.O. Box 11566 Cairo, Egypt; dalia.mahmoud@sci.asu.edu.eg (D.M.); magdaradi@sci.asu.edu.eg (M.R.); sara_alashaal@sci.asu.edu.eg (S.A.); 2 The Regional Centre for Mycology and Biotechnology (RCMB), AL- Azhar University, P.O. Box 11651 Cairo, Egypt; basmafarag.18@azhar.edu.eg; 3 Department of Clinical Pathology, Faculty of Medicine, Cairo University, P.O. Box 12613 Cairo, Egypt; miroislamic@yahoo.co.uk; 4 Department of Pediatrics, College of Medicine, King Saud University, King Khalid University Hospital, Medical City, P.O. Box 2925, Riyadh 11545, Saudi Arabia; talahmadi@ksu.edu.sa; 5 Department of Botany and Microbiology, College of Science King Saud University, P.O. Box 2455, Riyadh 11451, Saudi Arabia; sharbi@ksu.edu.sa

**Keywords:** Cockroaches, antimicrobial, multidrug-resistant (MDR), transmission electron microscope (TEM)

## Abstract

The insect gut is home to a wide range of microorganisms, including several bacterial species. Such bacterial symbionts provide various benefits to their insect hosts. One of such services is providing metabolites that resist infections. Little data are available about gut-inhabiting bacteria for several insect groups. Through the present work, the gut bacteria associated with the American cockroach (*Periplaneta americana* L.) were isolated, identified, and studied for their potential antimicrobial activity against multidrug-resistant (MDR) human pathogens. The cockroaches were collected from three different environmental sites. Gut bacteria were isolated, and sixteen species of bacteria were identified using Vitek MALDI-TOF MS. The antagonistic activity of the identified bacteria was tested against a panel of multidrug-resistant bacteria and fungi, namely: methicillin-resistant *Staphylococcus aureus* (MRSA) (clinical isolate), *Streptococcus mutans* Clarke (RCMB 017(1) ATCC ^®^ 25175™) (Gram-positive bacteria), *Enterobacter cloacae* (RCMB 001(1) ATCC^®^ 23355™) and *Salmonella enterica* (ATCC^®^ 25566™) (Gram-negative bacteria). The isolates were also tested against human pathogenic fungi such as *Candida albicans* (RCMB005003(1) ATCC^®^ 10231™), *Aspergillus niger* (RCMB002005), *Aspergillus fumigatus* (RCMB002008), *Aspergillus flavus* (RCMB002002), and *Penicillium italicum* (RCMB 001018(1) IMI193019). The results indicated that some bacterial species from the cockroach gut could antagonize the growth activity of all the tested pathogens. Such antimicrobial properties could ultimately lead to the future development of therapeutic drugs. The evaluation and mode of action of antagonistic gut bacteria against the most affected MDR pathogens were demonstrated using transmission electron microscopy (TEM).

## 1. Introduction

Drug-resistant pathogens are one of the most challenging issues facing public health today [1]. The WHO considers drug-resistant bacterium as one of the ten most threatening health issues confronting humanity in the present century [2]. Many species of bacteria have developed strong resistance against several groups of antimicrobial agents, especially with the rapid global spread of multi- and pan-resistant bacteria that have induced infections while not being treated with existing antimicrobial drugs [3]. In the same way, the problem of drug resistance is found in the treatment of pathogenic fungi. Searching for new sources of antimicrobial agents for tackling this problem is becoming increasingly urgent. Insects form one of these interesting new alternative sources of drugs for the next era [4].

Insects that live in polluted environments have a strong immune system for resisting microbial infections. The gut-inhabiting bacteria of such insect species produce antimicrobial agents that form a potential source of novel antimicrobial compounds [5,6]. Insects possess a wide variety of antagonistic bacteria that produce bioactive elements, which are being isolated and characterized for their ability in the treatment of some human diseases. As the insect gut is considered to be a very favorable niche for microbial colonization, symbiont bacteria potentially provide many beneficial services to their hosts [7]. Many insect species display a wide range of dependence on gut bacteria for some basic functions: some microorganisms protect their insect hosts against pathogens, parasitoids, and other parasites by synthesizing specific toxins that aid the insect immune system [8]. The gut microbiome of butterflies and moths (Lepidoptera), for example, shows a high variability between and within species. Gut bacteria of the families Enterobacteriaceae, Bacillaceae, and Pseudomonadaceae were the most widespread across lepidopteran species, with *Pseudomonas*, *Bacillus*, *Staphylococcus*, *Enterobacter*, and *Enterococcus* being the most common genera [9]. On the other hand, many insect groups have rarely been tested for their gut-inhabiting microorganisms, including the cockroach.

Cockroaches belong to the order Blattodea of the class Insecta. There are about 4000 known species worldwide. They are found everywhere, especially in bat caves, in human dwellings, under stones, on trees and plants, in forest litter. Most cockroach species are omnivorous. They feed mainly on plant sap, dead animals, and vegetable matter. From an ecological point of view, cockroaches play an important role in the environmental balance by digesting a wide range of waste substances, including decomposing forest and animal waste material. Although household cockroaches can contaminate food and can spread human diseases [10], their gut microbiota plays an important role in their own health and fitness [11,12,13]. Several associated bacterial families have been reported from cockroaches, particularly members of the family Enterobacteriaceae, but species of Staphylococcaceae and Mycobacteriaceae have also been found. *Streptomyces*, *Bacillus*, *Enterococcus*, and *Pseudomonas* were the most commonly reported genera from the cockroach gut [14]. In view of the wide range of cockroach habitats and of their species diversity, the gut-inhabiting bacteria of most of them are hardly known [15]. The American cockroach, *Periplaneta americana* L. (Blattidae), is one of the species for which we have insufficient knowledge of their gut microbiota [16].

*P. americana* is common in tropical climates, but human activity has extended its range, and it is now virtually cosmopolitan in distribution due to global commerce [17]. It is a pest that can threaten human health as it is the largest of the house-infesting roaches [18]. American cockroaches can contaminate food with bacterial pathogens that result in food poisoning, dysentery, and diarrhea, and these can cause childhood asthma [19]. Very little information about the gut bacterial symbionts of *P. americana* is available despite its widespread distribution [20]. Furthermore, as it lives in very polluted environments (e.g., garbage and drainage pipes), the gut-inhabiting symbionts of this species can be expected to show antimicrobial activity.

New antimicrobial alternatives are very important for sustaining the level of infection control through our public health systems [21]. There is a wide range of drug-resistant pathogens, including several species of bacteria and fungi. Among this multidrug-resistant (MDR) diversity, bacteria such as *Enterobacter cloacae* have emerged as a significant nosocomial pathogen in neonatal units, with numerous outbreaks of infection being reported [22,23]; *Salmonella enterica*, associated with typhoid and paratyphoid fever [24]; *Staphylococcus aureus*, a natural inhabitant of human and animal skin but sometimes able to cause infections affecting many organs and also incriminated in food poisoning [25]; *Streptococcus mutans*, the main contributor to tooth decay and oral infections [26]. These are all species that have become difficult to treat with conventional antibiotics [27]. On the other hand, there are pathogenic fungi that are among species that can resist a wide range of anti-fungal drugs [28]: *Aspergillus flavus*, which produces aflatoxin B1, the most toxic and potent hepatocarcinogenic natural compound ever found [29]; *A. fumigatus*, a species incriminated in a wide range of diseases including chronic pulmonary aspergillosis [30]; *A. niger*, known to produce mycotoxins called ochratoxins [31]; *Candida albicans*, which initiates a wide range of diseases such as chronic disseminated candidiasis, endocarditis, vaginitis, meningitis, and endophthalmitis [32]; *Penicillium italicum*, attributed to the pathogenesis of pneumonia, hypersensitivity, allergic alveoli, skin sensitivity, and emphysema [33].

Based on this, the originality of our work rests on the search for new alternative antimicrobial agents from insect sources by evaluating the antagonistic activity of bacteria isolated from the gut of *P. americana* L. against certain human pathogens. Four types of MDR bacteria (*Streptococcus mutans*, *Enterobacter cloacae*, *Staphylococcus aureus*, and *Salmonella enterica*) and three types of MDR fungi (*Aspergillus* spp., *Candida albicans*, and *Penicillium italicum*) were used to test the antipathogenic effects of *P. americana* gut symbionts.

## 2. Materials and Methods

### 2.1. Collection of Cockroaches and Extraction of Gut Bacteria

#### 2.1.1. Sample Collection

Adult American cockroaches, *P. americana* L., were collected from three different environmental sites in three different governorates: a paper factory (Cairo), a food store (Qalyubia), and sewage water (Giza), Egypt. Bait trapping and active collection methods were used [34]. The identification of samples was performed using the standard taxonomic key [35]. A total of 231 individuals were collected, while only some of them were used to perform the experiments.

#### 2.1.2. Isolation and Identification of the Gut-Associated Bacteria

Cockroaches were transferred to the laboratory of the Entomology Department, Faculty of Science, Ain Shams University, where their guts were removed using a sterile blade and forceps. The gut was completely crushed with a mortar in 1 mL sterile distilled water. 50–100 μL of the sample was inoculated into the nutrient agar medium. The inoculated samples were incubated at 37 °C for 24–48 h. Morphologically different colonies were selected. The collected samples were further sub-cultured on an appropriate medium. All the isolated samples were stored at 4 °C for 15–20 min, and the isolated colonies were subjected to the phenotypic analysis method, which includes analysis of color, consistency, surface texture, appearance, and opaqueness. All bacterial isolates were stained with Gram’s dye for the identification of Gram-positive or Gram-negative bacteria [36]. Finally, isolated gut bacteria were identified by matrix-assisted laser desorption ionization-time of flight mass spectrometry (Vitek MALDI-TOF MS), which depends on the protein profile and biochemical activities of each isolate [37]. The samples were performed in duplicate, with tests performed simultaneously on the same target slide. Part of a single colony was transferred to an individual spot on the 48-well Vitek Mass spectrometry disposable slides MS-DS. Each spot was covered with 1 µL ready-to-use Vitek MS alpha-cyano-4-hydroxycinnamic acid (HCCA) matrix (bioMérieux, France). The target plate was then read, the spectra were acquired using the MALDI-TOF Vitek MS (bioMérieux) and analyzed on Vitek Mass spectrometry in vitro diagnostic MS-IVD system (bioMérieux; Marcy l’Etoile, France). The protein profiles of each specimen with an m/z of 3000 to 15,000 were produced, and the profiles were further matched with the Vitek MS reference CE-IVD certified database (>20,000 spectra). Matching results with confidence percentages of 90% to 98% confidence were considered for genus level, results of >98% confidence were considered for species level, but <90% confidence was unacceptable for identification. All isolated and identified bacteria were given a number from 1 to 16, and we will refer to them according to these numbers throughout this article.

### 2.2. Antagonistic Activity of the Gut Associated Bacteria

Multidrug-resistant (MDR) bacteria and fungi were obtained from the Regional Center for Mycology and Biotechnology Antimicrobial Unit Test Organism, Al-Azhar University, Nasr City, Egypt. Gram-positive bacteria used were methicillin-resistant *Staphylococcus aureus* (MRSA) (clinical isolate), and *Streptococcus mutans* Clark (RCMB 017(1) ATCC ^®^ 25175™), and the Gram-negative bacteria used were *Enterobacter cloacae* (RCMB 001(1) ATCC^®^ 23355™) and *Salmonella enterica* (ATCC^®^. 25566™). Human pathogenic fungi used were *Candida albicans* (RCMB005003(1) ATCC^®^ 10231™), *A. niger* (RCMB002005), *A. fumigatus* (RCMB002008), *A. flavus* (RCMB002002), and *Penicillium italicum* (RCMB 001018(1) IMI193019). The purified colonies of isolated gut bacteria were cultured on nutrient broth medium, which was prepared by adding 13 gm of a mixture of beef Extract-Peptone-Sodium Chloride to 1 L of distilled water, which was mixed to dissolve completely and sterilized by autoclaving at 121 °C for 15 min; incubation was then done at 37 °C. for 24 h in a shaker incubator before testing their antagonistic activity [38]. The agar well diffusion method was used to test the antagonistic activity of the gut-isolated bacteria against the selected human pathogens (using nutrient agar media for testing bacteria and malt extract agar media for testing fungi) [39]. Then the plates were incubated for 24 h at 37 °C for testing human pathogenic bacteria and 28 °C for testing human pathogenic fungi. The zones of inhibition of pathogenic bacteria and fungi were measured by a transparent ruler. Three replicates for each test were done for every evaluated pathogenic species. Matrix cluster analyses using two-way single linkage Euclidian distance were made to evaluate the degree of antimicrobial activity for each bacterial symbiont. Statistical analysis was made using SYSTAT version 13, from Systat Software, Inc., San Jose, CA, USA, www.sigmaplot.com (accessed on 30 December 2020).

### 2.3. Transmission Electron Microscopy (TEM)

Morphological studies of the most affected pathogenic bacteria and fungi that were treated with isolated gut bacteria were demonstrated by TEM (JEOL 1010). For TEM preparation, the samples were fixed in 3% glutaraldehyde, rinsed in phosphate buffer, and post-fixed in potassium permanganate solution for 5 min at room temperature. The samples were dehydrated in an ethanol series ranging from 10% to 90% for 15 min in each alcohol dilution and finally with absolute ethanol for 30 min. Samples were infiltrated with epoxy resin and acetone through a graded series until finally in pure resin. Ultrathin sections were collected on copper grids. Sections were then double-stained in uranyl acetate, followed by lead citrate. Stained sections were observed with a JEOL-JEM 1010 transmission electron microscope at 70 kV at the Regional Center for Mycology and Biotechnology (RCMB), Al- Azhar University [40,41].

## 3. Results

### 3.1. Identification of Isolated Gut Bacteria by Using Vitek (MALDI-TOF MS)

Table 1 reveals that the bacteria isolated from the paper factory belonged mostly to the families Enterobacteriaceae (25%), Brucellaceae (8%), and Xanthomonadaceae (4%), while bacteria isolated from the food store belonged mostly to the families Enterobacteriaceae (15%), Comamonadaceae (2%) and Micrococcaceae (3%). Finally, the bacteria isolated from sewage water belonged mostly to the families Bacillaceae (29%), Enterobacteriaceae (27%), and Staphylococcaceae (2%). Most of the isolated gut bacteria belonged to the families Bacillaceae and Enterobacteriaceae.

All bacteria species were isolated from a single site except two species: *Bacillus sphaericus*, which was isolated from insects collected at the paper factory and from sewage water, referred to as 1 and 12, respectively; and *Serratia marcescens*, which was isolated from insects collected from the food store and from sewage water, referred to as 4 and 15.

### 3.2. Evaluating the Antagonistic Activity of Isolated Gut Bacteria

**Figure 1:**
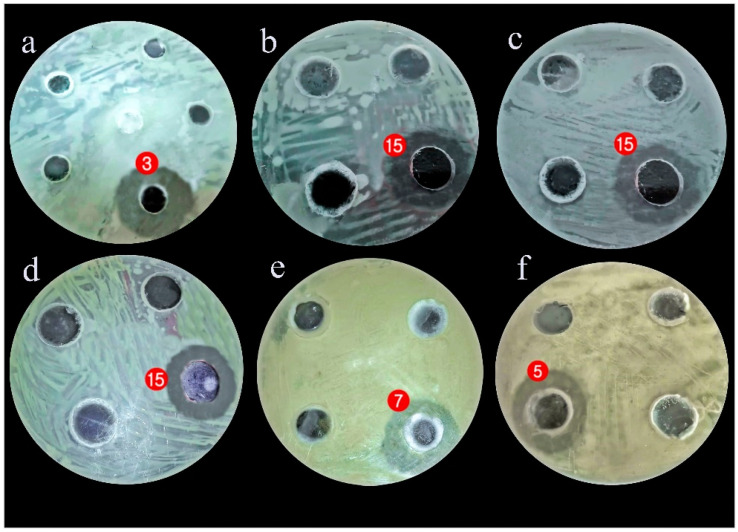
The inhibitory effect of (**a**) Stenotrophomonas maltophilia (3) against Streptococcus mutans, (**b**) Serratia marcescens (15) against Streptococcus mutans; (**c**) Serratia marcescens (15) against Enterobacter cloacae; (**d**) Serratia marcescens (15) against MRSA; (**e**) Bacillus cereus (7) against Candida albicans; (**f**) Delftia acidovorans (5) against Penicillium italicum.

In measuring the antagonistic activity of isolated gut bacteria against a panel of selected MDR human pathogens, it was found that most of them produced an inhibitory effect against the tested pathogens, and the measured inhibition zones are listed in Table 2 and Table 3 and Figure 1.

Table 2 provides information about the antagonistic activity of isolated gut bacteria against the tested microbes. *Stenotrophomonas maltophilia* had the highest antagonistic growth effect against *Streptococcus mutans* (37 ± 0.3 mm) while *Serratia marcescens* (15) had a high multiple antagonistic effects against the growth of *Streptococcus mutans* with an inhibition zone of 35 ± 0.1 mm, MRSA with an inhibition zone of 30 ± 0.1 mm, and *Enterobacter cloacae* with an inhibition zone of 20 ± 0.2 mm. Gram-positive bacteria (*Streptococcus mutans* and MRSA) were more sensitive than Gram-negative bacteria (*Enterobacter cloacae* and *Salmonella enterica*).

Table 3 shows that seven isolated bacteria had inhibitory activity against the tested fungi. *Bacillus cereus* has the highest antagonistic effect against *Candida albicans* (25 ± 0.4 mm). Also, *Serratia marcescens* (15) shows multiple antagonistic effects against *Aspergillus niger* and *Candida albicans* with inhibition zones of 13 ± 0.4 mm and 11 ± 0.5 mm, respectively, while *Delftia acidovorans* induced good inhibition against *Penicillium italicum* and *A. fumigatus*, with 23 ± 0.5 mm and 20 ± 0.4 mm inhibition zones, respectively.

**Figure 2:**
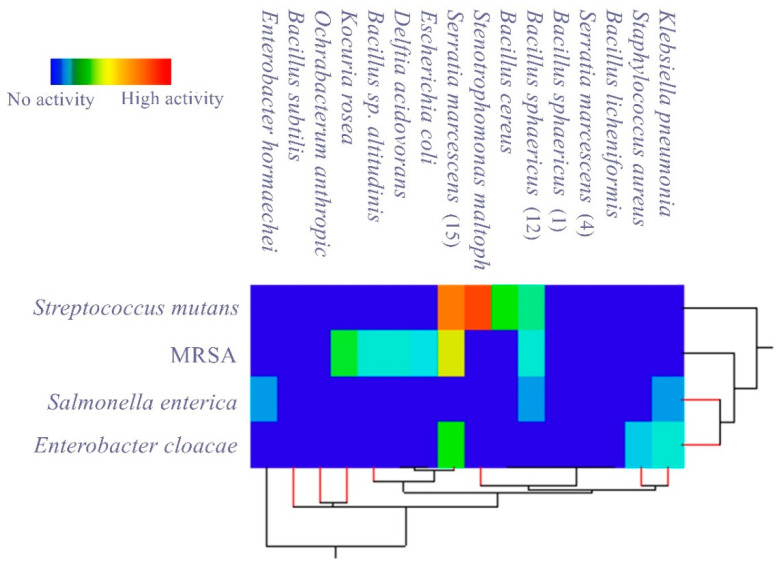
Matrix cluster analysis based on two-way single linkage Euclidian distance showing the level of antagonistic activity of bacterial symbionts isolated from *P. americana* L. against tested MDR pathogenic bacteria.

**Figure 3:**
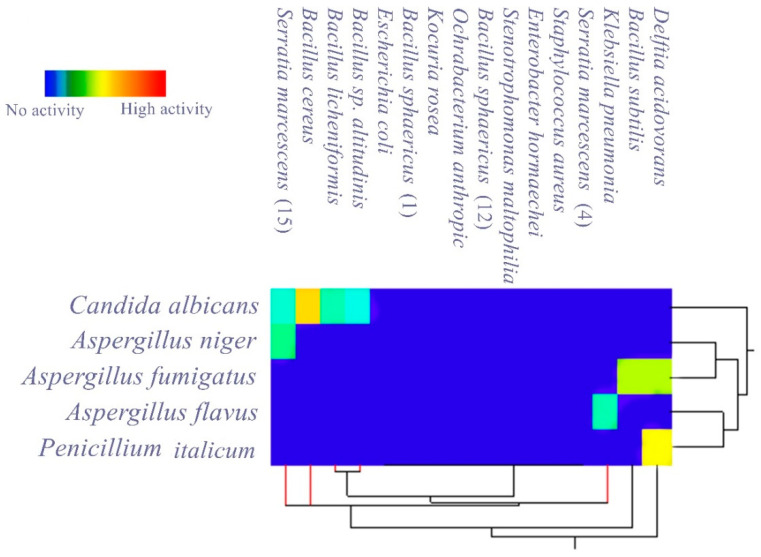
Matrix cluster analysis based on two-way single linkage Euclidian distance showing the level of antagonistic activity of bacterial symbionts isolated from *P. americana* L. against tested MDR pathogenic fungi.

Matrix cluster analysis indicated a wide degree of antagonistic activity of bacterial symbionts, from no activity to highly active. For the pathogenic bacteria (Figure 2), the highest activity was reported from *Stenotrophomonas maltophilia* against *Streptococcus mutans*, followed by *Serratia marcescens* (15) against *Streptococcus mutans* and MRSA. With the pathogenic fungi (Figure 3); however, the bacterial symbionts showed less antagonist activity toward fungi than against bacteria. The highest activity was shown by *Bacillus cereus* against *Candida albicans*.

### 3.3. Ultrastructural Changes Shown by the Tested Pathogens Due to the Antagonistic Effects of Bacterial Symbionts

**Figure 4:**
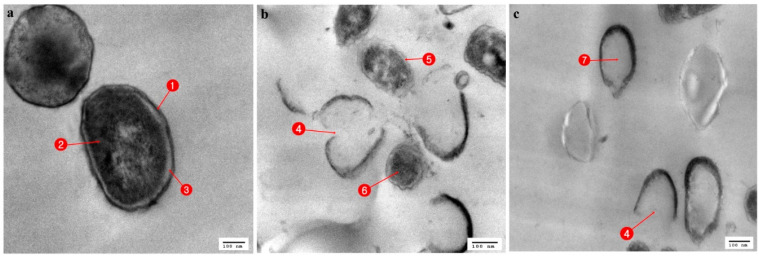
TEM micrograph of *Streptococcus mutans*; (**a**) Normal cells: 1. Cell wall, 2. Homogenous cytoplasm, 3. Cell membrane; (**b**) cell affected with *Stenotrophomonas maltophilia*: 4. Cell rupture, 5. Damage of cell wall and membrane, 6. Leakage of cytoplasm; (**c**) cell affected with *Serratia marcesens* (15): 7. Empty cell.

**Figure 5:**
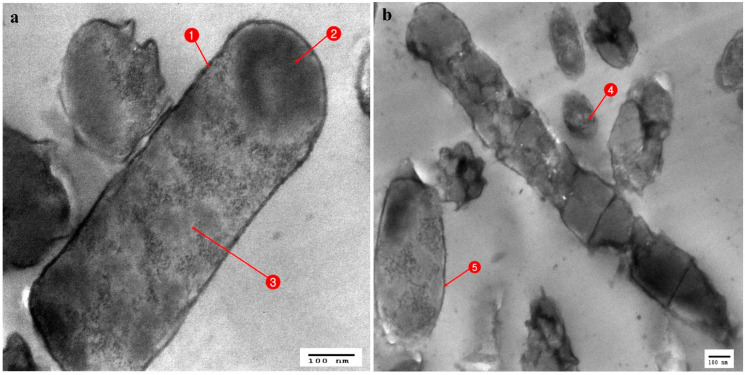
TEM micrograph of *Enterobacter cloacae*; (**a**) Normal cells: 1. Cell wall, 2. Nucleic acid material, 3. Uniform cytoplasm; (**b**) cell affected with *Serratia marcesens*: 4. Precipitation with cytoplasm, 5. Rigid and irregular cell wall.

**Figure 6:**
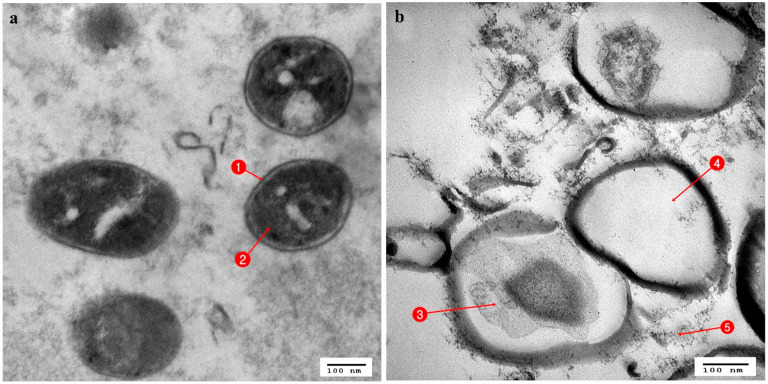
TEM micrograph of MRSA; (**a**) Normal cells: 1. Cell wall; 2. Cytoplasm; (**b**) cell affected with *Serratia marcesens*: 3. Shrinkage of cytoplasm; 4. Empty cell; 5. Leakage of cell material.

Regarding the TEM stained ultrathin sections (70 nm) of all tested organisms, in *Streptococcus mutans* ultrathin sections, the control cells showed an identical spherical (coccoidal) shape (Figure 4a), but when treated with *Stenotrophomonas maltophilia* (Figure 4b) and *Serratia marcescens* (15) (Figure 4c) showed great alterations in their cell structure. This resulted in membrane damage which appeared to have the potential to rupture, with leaks of intracellular materials and cellular damage finally leading to complete cell deformation. When the control cells of *Enterobacter cloacae*, which showed a rod-shaped structure with an undamaged and intact outer membrane (Figure 5a), were compared with the affected cells treated with *Serratia marcescens* (15), it appeared that all cells were lysed and devoid of cytoplasmic fluid, with complete shrinkage of the cytoplasmic membrane (Figure 5b). Untreated cells of *Staphylococcus aureus* (MRSA) had a normal cell condition, with a spherical shape and rigid surface with cytoplasm, continuously in close contact with the cell wall with normal intact cytoplasmic membrane (Figure 6a). Complete cellular damage, with breaks in the cell wall and leaks of cytoplasmic materials, was observed after treatment with *Serratia marcescens* (15), which is illustrated in Figure 6b.

**Figure 7:**
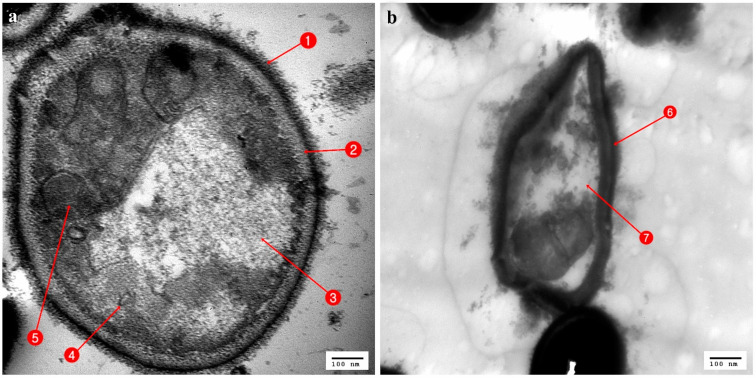
TEM micrograph of *Candida albicans* (**a**) Normal cells: 1. Cell wall; 2. Cell membrane; 3. Vacuole; 4. Mitochondria; 5. Nucleus; (**b**) cell affected with *Bacillus cereus*; 6. Irregular cell wall; 7. Nonhomogeneous cytoplasm.

The normal *Candida albicans* showed rounded cells with an intact cell wall (CW), cell membrane (CM), mitochondria (M), nucleus (N), and vacuole (V) (Figure 7a). In contrast, ultrathin sections of *Candida albicans* treated with *Bacillus cereus* revealed interference activity upon the structure of the yeast with alterations to the cell wall, which became irregular, and with changes in the cytoplasmic membrane, which increased in thickness and showed cytoplasmic materials constricted in the center of the cell (Figure 7b).

**Figure 8:**
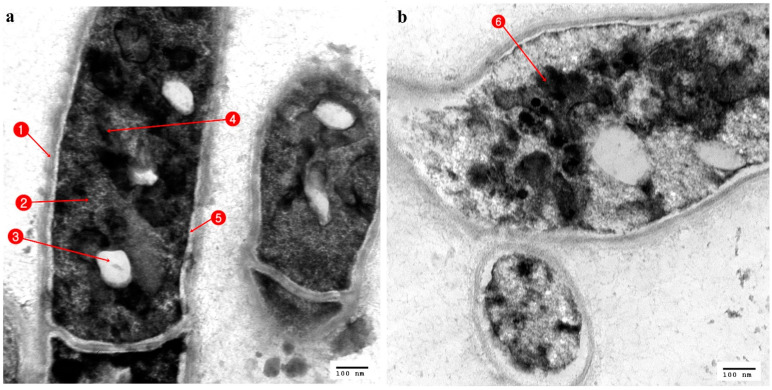
TEM micrograph of *Penicillium italicum*; (**a**) Normal cells; 1. Cell wall; 2. Nucleus; 3. Vacuole; 4. Mitochondria; 5. Cell membrane; (**b**) cell affected with *Delftia acidovorans*; 6. Nonhomogeneous cytoplasm.

Compared to the control ultrastructure of *Penicillium italicum* (Figure 8a), with an intact cell wall (CW) and cell membrane (CM) with identified organelles, mitochondria (M), vacuole (V), and nucleus (N), the *Penicillium italicum* treated with *Delftia acidovorans*, showed deformations of both the cell wall and cell membrane, with the disappearance of the identified cytoplasmic materials, which were concentrated in the center of the cell, and there was the appearance of numerous vacuoles (Figure 8b).

## 4. Discussion

The rapid emergence of antibiotic resistance among human and animal pathogens represents a major health threat. It is projected that by 2050, infections from antibiotic-resistant pathogens will exceed ten million cases [15,42,43,44], which will increase the cost of treatment and require longer hospitalizations. The emergence of this resistance to most conventional antibiotics has forced researchers to look for new promising alternatives. Testing antimicrobial agents from different natural sources such as plants, insects, and sometimes animals can lead to the discovery of new powerful substitutional antimicrobial drugs [45,46].

Many researchers have suggested that the microbial gut flora of insects living in polluted habitats such as cockroaches could produce certain antagonistic metabolites to resist microbial infections. Even insects that inhabit unpolluted environments (e.g., bees and wasps) have a gut microflora that has antagonistic effects against pathogens [47]. Miroslava et al. 2020 isolated and identified the gut bacteria of *Apis mellifera* and evaluated its antagonistic effect against *Paenibacillus* larvae, which causes American foulbrood (AFB) in honeybees [48]. The results of Guzman and Vilcinskas [15] revealed that the gut bacteria of cockroaches produce active molecule(s) with potent antibacterial properties. In their specific niches, microorganisms compete for nutrients and space by producing defensive molecules. These molecules gave a growth advantage to producer species by killing or inhibiting the growth of other species. Producer species are usually immune to these molecules [49].

Our results have shown that the variation in types of bacterial loads in cockroaches, as well as their pathogenicity, are related to their environmental heterogeneity [50,51,52]. Douglas, in 2015, indicated that, in general, insect microbiota differs from microorganisms in the external world, including food that is ingested, but that the conditions and resources in the insect habitat favor certain microbial taxa with certain characteristics that could be different from the characteristics of the same species in other habitats [53,54]. Such a case fits with our findings, as the strains of *Bacillus sphaericus* (12) and *Serratia marcescens* (15), which were isolated from cockroaches collected from sewage systems-a highly polluted environment-showed a high antagonistic activity against pathogenic organisms, while the different strains of the same species *Bacillus sphaericus* (1) and *Serratia marcescens* (4), which were isolated from cockroaches from less polluted environments, showed no antagonistic activity at all.

The matrix cluster analysis indicated that there were three bacterial isolates from the American cockroach with satisfactory antagonistic activity against the tested pathogens and are promising for discovering new antimicrobial drugs: *Stenotrophomonas maltophilia*, which had relatively high inhibition activity against *Streptococcus mutans*; *Serratia marcescens* (15), which antagonized the growth of three important drug-resistant pathogens (*Streptococcus mutans*, *Enterobacter cloacea*, and MRSA); *Bacillus cereus*, which had antagonistic activity against *Candida albicans*. On the other hand, *Bacillus sphaericus* had only a mild antagonistic activity against *Streptococcus mutans*, MRSA, and *Salmonella enterica*.

Several previous studies have indicated the antimicrobial effects of some identified bacterial symbionts isolated from cockroaches. For example, *Stenotrophomonas maltophilia* showed antimicrobial activity against the phytopathogens *Rhizoctonia solani* and *Pythium ultimum* [55]. Our results also agree with those of Berg [56], who recorded that *S. maltophilia* inhibited the growth of *R. solani*, possibly because of antibiosis and the production of some lytic enzymes that act against pathogenic fungi. In addition, another study recorded an apparent reduction in the growth of *R. solani* exposed to volatile molecules produced by *S. maltophilia* [57,58].

Bacteria have different mechanisms to support their antagonistic activity against other microorganisms, such as the production of toxins, peptides, antibiotics, bacteriocins, and enzymes that interfere with the growth of other microbial competitors. Bacteriocins are proteinaceous or peptidic toxins produced by some species of bacteria to inhibit the growth of similar or closely related bacterial strains and are structurally, functionally, and ecologically diverse. The applications of some bacteriocins are being tested to assess their application as narrow-spectrum antibiotics [59]. The chemistry behind the results obtained from our work, especially those from the electron microscope, could indicate the presence of some types of bacteriocins that, if identified, could help to produce new effective antibiotics against MDR microbes in the near future. Natural products derived from insect–microbial symbioses have vast biochemical properties that suggest that they could provide promising drugs, especially against difficult-to-treat microbial infections [60].

Concerning pathogenic fungi, the gut bacterium *Delftia acidovorans* recorded a high antagonistic activity against *Aspergillus fumigatus*, which is a pathogenic fungus that is the most common etiological agent of human aspergillosis, and *Penicillium italicum*, which is a known causative agent of necrotizing esophagitis, endophthalmitis, keratitis, and asthma [61]. The nucleus of *Penicillium italicum* became empty due to the nuclease activity of *Delftia acidovorans*, and such activity could be directed against the nuclear chromosomal DNA, a mechanism that was hypothesized by Gautam et al. [62].

*Serratia marcescens* (15) also demonstrated remarkable inhibitory influence against the pathogenic fungus *Aspergillus niger*, which is known to produce certain mycotoxins that are hepatocarcinogenic and nephrogenic immunological in nature [28,63], and against *Candida albicans* which under certain conditions can initiate a wide range of diseases such as chronic disseminated candidiasis, endocarditis, vaginitis, meningitis, and endophthalmitis [31]. The gut bacteria *Klebsiella pneumonia* is the only isolated bacterium from all the isolated bacteria that showed antigenicity toward *Aspergillus flavus*, which is considered to be the second leading cause of invasive aspergillosis and is the most common cause of superficial infection [64]. A *Serratia marcescens* strain, previously isolated from the citrus rhizosphere in São Paulo State, had its antagonistic activity against *Phytophthora parasitica* established [65], which explained the inhibition of fungal growth in KB media due to the production of siderophores as well as antibiotics from associated *Serratia* sp.

The Bacillaceae group (*Bacillus subtilis*, *Bacillus cereus*, and *Bacillus licheniformis*) proved its antagonistic potential against *Aspergillus fumigatus* and *Candida albicans*, respectively. *Bacillus subtilis* which was isolated from a pepper stem, confirmed its antimicrobial activity against the pathogenic fungi *Fusarium solani*, *Sclerotium rolfsii*, *Rhizoctonia solani*, and *Erwina carotovora* [66,67]. *Bacillus subtilis* is known to produce antibiotics such as iturins and bacillomycins as part of its’ antimicrobial activities [47].

*Serratia marcescens* (15) and *Stenotrophomonas maltophilia* showed promising antagonistic activity against most tested pathogenic microbes. Antagonism was confirmed by ultrastructural changes observed in tested pathogens. The cell-leakage was assumed to be due to alterations in the cell wall permeability of the pathogen cells, leading to pore formation in the plasma membrane, which, in turn, led to the deformation of ion exchange channels. Such a mechanism is due to the action of bacteriocins, and this is supported by [61,68]. Shrinkage of the cytoplasm in Figure 4 (MRSA) following treatment by *Serratia* was assumed to be induced by the efflux of phosphate or K+, causing the depletion of cytoplasmic ATP [69].

Finally, of the 16 types of identified gut bacteria, we identified only four species that were previously isolated and identified from the guts of different species of cockroach (*Serratia marcescens*, *Klebsiella pneumonia*, *Escherichia coli*, and *Bacillus* sp.). Further studies are therefore needed to characterize the chemical nature involved in the inhibitory activities of such bacteria. The present findings suggest that some bacterial species associated with *P. americana* L. could be used to develop various pathogen management strategies until new pharmaceutical products are developed. A large-scale study to screen the bacterial inhabitants of the *P. americana* L. gut from different geographic locations and/or at different seasons would therefore be useful in the search for new biological agents that could potentially be applied to the discovery of more antimicrobial agents. An understanding of the bacteriome inhabiting the intestine of different species of cockroaches could help in the development of advanced antibiotics that resolve the problem of drug-resistant microbes.

## 5. Conclusions

The present work showed that some of the gut bacteria isolated from the American cockroach, *P. americana* L., had outstanding antimicrobial activities against the most tested MDR human pathogens. It is evident that the isolated gut bacteria could produce novel compounds (metabolites) that may be used as substitutes for current antimicrobial drugs in order to overcome the problem of drug resistance. Our work will pave the way for the identification of new antimicrobial compounds from the gut microbes of the American cockroach, *P. americana* L., especially of the secondary metabolites of the enteric bacterium *Serratia marcescens*, as it revealed a high level of antagonistic activity against a high percentage of multidrug-resistant human pathogens, similar to the secondary metabolites of *Delftia acidovorans* and *Stenotrophomonas maltophilia*. Bacteriocins of *Delftia acidovorans* are also substitutes for overcoming bacterial and fungal infections. Of the 16 bacterial species isolated through this study, only four species were recorded previously from cockroaches, while the others were isolated and identified for the first time from the American cockroach *P. americana* L., which were here considered to be a newly-recorded host for them.

## Figures and Tables

**Table 1:** Summarizing list of isolated gut bacteria of *Periplaneta americana* L. from the three collecting sites and their frequency.

Environmental Sites	Identification of Gut Bacteria	Frequency of Occurrence
Paper Factory	*Bacillus sphaericus* *	25%
	*Ochrabactrum anthropi*	8%
	*Stenotrophomonas maltophilia*	4%
Food store	*Serratia marsescens* **	15%
	*Delftia acidovorans*	2%
	*Kocuria rosea*	3%
Sewage Water	*Bacillus cereus*	4%
	*Enterobacter hormaechei*	8%
	*Bacillus subtilis*	2%
	*Bacillus sp. altitudinis*	20%
	*Bacillus licheniformis*	3%
	*Bacillus sphaericus* *	-
	*Staphylococcus aureus*	2%
	*Escherichia coli*	2%
	*Serratia marcescens* **	-
	*Klebsiella pneumonia*	2%
Total no. of bacterial isolates = 100		100%

* *Bacillus sphaericus*: isolated from two different environmental sites (paper factory and sewage water). ** *Serratia marcescens*: isolated from two different environmental sites (food store and sewage water).

**Table 2:** Antagonistic activity of isolated gut bacteria against multidrug-resistant (MDR) Gram-positive and Gram-negative bacteria.

Isolated Gut Bacteria		Growth Inhibition Zone (mm)			
	Pathogenic Bacteria	*Streptococcus mutans.*	*Enterobacter cloacae*	MRSA	*Salmonella enterica*
*Bacillus sphaericus*		-	-	-	-
*Ochrabacterum anthropi*		-	-	-	-
*Stenotrophomonas maltophilia*		37 ± 0.3	-	-	-
*Serratia marcescens*		-	-	-	-
*Delftia acidovorans*		-	-	12 ± 0.5	-
*Kocuria rosea*		-	-	18 ± 0.4	-
*Bacillus cereus*		20 ± 0.5	-	-	-
*Enterobacter hormaechei*		-	-	-	8 ± 0.3
*Bacillus subtilis*		-	-	-	-
*Bacillus sp. altitudinis*		-	-	12 ± 0.3	-
*Bacillus licheniformis*		-	-	-	-
*Bacillus sphaericus*		15 ± 0.3	-	12 ± 0.3	8 ± 0.2
*Staphylococcus aureus*		-	10 ±0.1	-	-
*Escherichia coli*		-	-	11 ± 0.2	-
*Serratia marcescens*		35 ± 0.1	20 ± 0.2	30 ± 0.1	-
*Klebsiella pneumonia*		-	12 ± 0.6	-	8 ± 0.3

The numbers represent means ± standard deviations. (-) absence of susceptibility.

**Table 3:** Antagonistic activity of isolated gut bacteria against MDR fungi.

Isolated Gut Bacteria		Growth Inhibition Zone (mm)				
	Pathogenic Fungi	*A. niger*	*A. fumigatus*	*C. albicans*	*P. iticulum*	*A. flavus*
*Bacillus sphaericus*		-	-	-	-	-
*Ochrabacterium anthropi*		-	-	-	-	-
*Stenotrophomonas sp.*		-	-	-	-	-
*Serratia marcescens*		13 ± 0.4	-	11 ± 0.5	-	-
*Delftia acidovorans*		-	20 ± 0.4	-	23 ± 0.5	-
*Kocuria rosea*		-	-	-	-	-
*Bacillus cereus*		-	-	25 ± 0.4	-	-
*Enterobacter hormaechei*		-	-	-	-	-
*Bacillus subtilis*		-	20 ± 0.3	-	-	-
*Bacillus sp. altitudinis*		-	-	10 ± 0.4	-	-
*Bacillus licheniformis*		-	-	12 ± 0.3	-	-
*Bacillus sphaericus*		-	-	-	-	-
*Staphylococcus aureus*		-	-	-	-	-
*Escherichia coli*		-	-	-	-	-
*Serratia marcescens*		-	-	-	-	-
*Klebsiella pneumonia*		-	-	-	-	12 ± 0.4

The numbers represent means ± standard deviations. (-) absence of susceptibility.
